# Enhancing predictive validity of motoric cognitive risk syndrome for incident dementia and all-cause mortality with handgrip strength: insights from a prospective cohort study

**DOI:** 10.3389/fnagi.2024.1421656

**Published:** 2024-06-21

**Authors:** Weimin Bai, Ruizhu Ma, Yanhui Yang, Juan Xu, Lijie Qin

**Affiliations:** ^1^Department of Emergency, Henan Provincial People’s Hospital, People’s Hospital of Zhengzhou University, People’s Hospital of Henan University, Zhengzhou, China; ^2^Department of Endocrinology, The People’s Hospital of Danyang, Danyang Hospital of Nantong University, Danyang, Jiangsu, China; ^3^Department of Cardiology, The Second Medical Center, Chinese PLA General Hospital, Beijing, China; ^4^Department of General Surgery, Affiliated Xiaoshan Hospital, Hangzhou Normal University, Hangzhou, China

**Keywords:** motoric cognitive risk syndrome, all-cause mortality, handgrip strength, Cox regression, net reclassification indices, integrated discrimination improvement

## Abstract

**Background:**

This study aimed to assess whether integrating handgrip strength (HGS) into the concept of motoric cognitive risk (MCR) would enhance its predictive validity for incident dementia and all-cause mortality.

**Methods:**

A cohort of 5, 899 adults from the Health and Retirement Study underwent assessments of gait speed, subjective cognitive complaints, and HGS were involved. Over a 10-year follow-up, biennial cognitive tests and mortality data were collected. Cox proportional hazard analyses assessed the predictive power of MCR alone and MCR plus HGS for incident dementia and all-cause mortality.

**Results:**

Patients with MCR and impaired HGS (MCR-HGS) showed the highest adjusted hazard ratios (AHR) for dementia (2.33; 95% CI, 1.49–3.65) and mortality (1.52; 95% CI, 1.07–2.17). Even patients with MCR and normal HGS (MCR-non-HGS) experienced a 1.77-fold increased risk of incident dementia; however, this association was not significant when adjusted for socioeconomic status, lifestyle factors, and medical conditions. Nevertheless, all MCR groups demonstrated increased risks of all-cause mortality. The inclusion of HGS in the MCR models significantly improved predictive discrimination for both incident dementia and all-cause mortality, as indicated by improvements in the C-statistic, integrated discrimination improvement (IDI) and net reclassification indices (NRI).

**Conclusion:**

Our study underscores the incremental predictive value of adding HGS to the MCR concept for estimating risks of adverse health outcomes among older adults. A modified MCR, incorporating HGS, could serve as an effective screening tool during national health examinations for identifying individuals at risk of dementia and mortality.

## Introduction

The global increase in both the elderly population and life expectancy has led to a significant rise in dementia cases, presenting a substantial public health challenge. Approximately 55 million people worldwide are affected by dementia, with the economic impact estimated at 1.1% of the global gross domestic product, a figure that is expected to double by 2030 (WHO, 2023). Projections indicate that the number of people living with dementia will rise from 55 million in 2019 to 139 million by 2050, with associated costs likely to surpass $2.8 trillion annually by 2030 (Alzheimer’s Disease International, 2023). In light of these projections, enhancing our understanding of dementia risk factors is essential, particularly through prospective, population-based studies. Although modifiable risk factors such as body mass index, alcohol consumption, smoking, poor diet, and physical activity have been linked to dementia (Livingston et al., 2020), data on markers of cognitive and physical capability, including subjective cognitive decline (Slot et al., 2019), gait speed (Dumurgier et al., 2017), muscular strength (Carson, 2018), or their combination (Chen et al., 2012; Montero-Odasso et al., 2020), are still limited.

Motoric cognitive risk syndrome (MCR) is a predementia condition characterized by both slow gait and subjective cognitive decline in elderly individuals without dementia (Verghese et al., 2013). Studies have shown that MCR prevalence varies from 2 to 18% in different countries (Congcong and Linping, 2021), with a pooled prevalence of 9.7% among individuals aged 60 and older across 17 countries (Verghese et al., 2014). MCR is linked to an increased risk of multiple falls (RR 1.77, 95% CI 1.25, 2.51) (Callisaya et al., 2016), incident dementia (Verghese et al., 2014; Beauchet et al., 2020), disability (Chhetri et al., 2017) and all-cause mortality (Bortone et al., 2022; Pajuelo-Vasquez et al., 2023). These findings highlight that MCR involves both cognitive and mobility impairments, posing challenges for families and healthcare systems. While slow gait in older adults has multiple causes (Camicioli et al., 1998; Verghese et al., 2007), including cognitive complaints in the MCR criteria improves its predictive validity (Verghese et al., 2014). Although informant reports can help in identifying dementia, their reduced sensitivity might overlook solitary older adults, thus narrowing the group of older adults considered at risk. The variability in the criteria for MCR is balanced by mutual enhancements, making MCR a more effective predictor of cognitive decline than either slow gait or cognitive complaints alone.

Physical capability, also known as physical functioning, describes an individual’s ability to perform daily physical tasks. Objective measures such as handgrip strength (HGS), walking speed, chair rising, and standing balance are not only indicators of physical capability but also markers for current and future health outcomes (Cooper et al., 2011), including all-cause mortality (Cooper et al., 2010). However, research into physical capability and dementia faces challenges including small sample sizes, short follow-up periods, and inadequate adjustment for confounding factors. Additionally, previous studies have shown the incremental predictive power of including chair rising or standing balance tests in established MCR frameworks (Sekhon et al., 2019b; Chung and Byun, 2023), but their prognostic value for all-cause mortality has not been fully explored. Muscle strength, especially HGS, is a valuable marker of wellbeing, associated with the ability to perform activities of daily living (ADLs). In the context of MCR, which involves subjective cognitive concerns and a slowing of gait speed while maintaining independence in basic ADLs, a decline in HGS might be overlooked without careful attention to MCR patients. HGS, assessed using a hand-held dynamometer, is favored for its simplicity, reliability, and cost-effectiveness, making it a preferred method in epidemiological studies. Several studies have demonstrated that HGS is an effective screening tool for predicting adverse outcomes and mortality in middle-aged and elderly populations (Bohannon, 2008; Cooper et al., 2010), as well as in very old community-dwelling populations (Ling et al., 2010). Furthermore, recent studies suggest that HGS may indicate brain health and cognitive decline (Alfaro-Acha et al., 2006; McGrath et al., 2019), although these associations could be prone to reverse causation bias. Further research into the links between HGS, MCR, and adverse health outcomes in large-scale population studies is needed to clarify its potential prognostic value.

The relationship between HGS, MCR, cognitive function, and gait in older adults has increasingly attracted scholarly interest. Jia et al. (2023) identified a strong link between HGS and MCR, suggesting that early identification of HGS asymmetry and decline might facilitate the prevention and treatment of MCR. Similarly, Zhang et al. (2020) found a negative correlation between HGS and the prevalence of MCR in older men, noting that more significant reductions in HGS were associated with an increased risk of MCR. Although various subtypes of MCR have been identified based on quantitative gait parameters (Allali et al., 2016) or cognitive subdomains (Bortone et al., 2022), the potential of HGS to predict future all-cause dementia and mortality has not yet been investigated. Moreover, to the best of our knowledge, no studies have concurrently assessed whether MCR or MCR-HGS estimates dementia and all-cause mortality in a large, nationwide, community-based population, or whether MCR patients with normal HGS have improved predictive accuracy over using MCR alone.

Addressing these research gaps, we proposed a modified MCR framework that incorporates both MCR and HGS, based on well-established criteria, and utilized data from a prospective cohort of community-dwelling older adults without dementia. We investigated the concurrent validity of MCR and MCR-HGS in predicting incident dementia and all-cause mortality. Our analysis also examined whether this modified MCR framework, including HGS, offers additional predictive value for incident dementia and all-cause mortality compared to using MCR alone in this nationwide cohort study.

## Materials and methods

### Sample

This study utilized data from Waves 10–15 of the Health and Retirement Study (HRS), a comprehensive longitudinal study exploring the aging process in Americans aged 51 and older. The HRS employs a multi-stage probability sampling method to achieve a nationally representative sample of this demographic (Heeringa and Connor, 1995). It gathers self-reported information on demographics, chronic health conditions, daily activities, disability status, and other health determinants initially and biennially thereafter. Starting in 2006, the HRS introduced an enhanced face-to-face interview including physical performance tests, biomarker collections, and a leave-behind questionnaire on psychosocial issues. In 2006, half of the households were randomly selected for the enhanced interview, with the remaining households included in 2008, a method maintained in later waves. Additional details on the HRS’s recruitment tactics and structure are provided in earlier publications (Heeringa and Connor, 1995).

**FIGURE 1:**
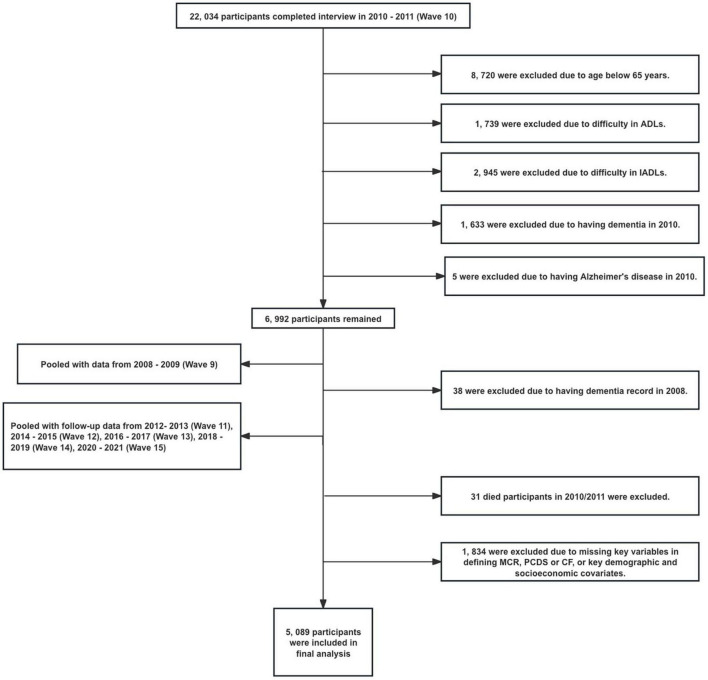
Study flowchart of participant selection.

The baseline analysis merged data from the 2008–2009 (Wave 9) and 2010–2011 (Wave 10) cycles, the first time participants were queried about Alzheimer’s disease (AD) or dementia, replacing previous questions about “memory-related disease.” Mortality information has been available since 2011. A total of 22,034 participants completed Wave 10 and were tracked biennially until 2020–2021 (Wave 15). The University of Michigan Institutional Review Board approved the HRS study. The final sample included 5,089 individuals who were 65 years or older, had comprehensive baseline data on MCR measures, reported no difficulties with ADLs or instrumental activities of daily living (IADLs) at baseline, were not diagnosed with AD or dementia initially, and were alive in 2010/2011. Figure 1 illustrates the flow of participants through each stage of selection based on these criteria.

### Measures

#### Motoric cognitive risk syndrome

MCR syndrome was defined by the presence of subjective cognitive complaints and slow gait in older adults who did not have a mobility disability or dementia (Verghese et al., 2012, 2013, 2014). In the HRS, gait speed, measured in meters per second, was determined by the time it took to walk a 2.5-meter course at a normal pace within participants’ homes. Slow gait was defined as performance at least one standard deviation (SD) below the age and sex-adjusted mean, a criterion previously used in the HRS to define MCR (Ayers and Verghese, 2016). Details of the cut-off points for slow gait were provided in Supplementary Table 1.

Subjective cognitive complaints were assessed using two questions: 1. “How would you rate your memory at the present time? Would you say it is excellent, very good, good, fair, or poor?” and 2. “Compared with the previous interview, would you say your memory is now better, about the same, or worse than it was?” Responses of “fair” or “poor” to the first question, or “worse” to the second, were used to identify cognitive complaints.

#### Handgrip strength

Muscle strength was evaluated using the average of two handgrip strength (HGS) measurements with a dynamometer on the dominant hand. The Smedley spring-type handgrip dynamometer (Scandidact; Odder, Denmark) was utilized for this purpose. Prior to testing, trained interviewers explained the HGS protocols and adjusted the dynamometer to fit the hand size of each participant. A practice trial was conducted with the participant’s arm positioned at the side and the elbow flexed at 90 degrees. Following the identification of the dominant hand, participants were instructed to squeeze the dynamometer with maximal effort, starting with the non-dominant hand. HGS was measured twice on each hand, alternating between hands. Participants who were unable to stand or position their arm while grasping the dynamometer were allowed to be seated and rest their upper arm on a supporting object during the HGS testing. Further details on the HGS measurement protocol in the HRS are available elsewhere (Crimmins et al., 2016). Weakness was identified when grip strength fell below thresholds adjusted for Body Mass Index (BMI) and gender, as established in the Cardiovascular Health Study (CHS) (Fried et al., 2001). Details of the criteria for weakness definition were also provided in Supplementary Table 1. Participants were noted as having missing data for physical measures if they were unable to perform the assessments due to lack of appropriate facilities or equipment, or due to recent surgery.

#### Dementia

Biennial cognitive function tests were administered by trained HRS interviewers either in-person or via telephone using the Modified Telephone Interview for Cognitive Status (TICS-m), which is a global cognition test based on the Mini-Mental State Examination. The TICS-m includes immediate and delayed 10-noun free recall tests (score range: 0–10 for each), a serial seven subtraction test (score range: 0–5), and a counting backward from 20 test (score range: 0–2). Higher scores indicate better cognitive performance. During each assessment, HRS participants were classified as having normal cognition, mild cognitive impairment (MCI), or dementia based on established thresholds and comprehensive evaluations, including expert clinician adjudication from the Aging, Demographics, and Memory Study (ADAMS), a dementia sub-study within the HRS framework. The diagnosis of dementia was based on physician-diagnosed dementia and TICS scores between 0 and 6 (Langa et al., 2005; Crimmins et al., 2011).

#### All-cause mortality

Mortality data were collected, including the year and month of death, sourced from an exit interview or the core interview of a spouse or partner.

### Statistical analysis

We assessed differences between the non-MCR groups, MCR patients with normal handgrip strength (MCR-non-HGS), and MCR patients with impaired handgrip strength (MCR-HGS) using a two-sided, independent *t*-test and the χ^2^ test. To evaluate the impact of MCR and MCR-HGS on all-cause dementia and mortality, Cox proportional hazards regression analysis was employed. The observation period ranged from the index date to the earliest of the following events: onset of dementia, death, or the end of the observation period on 31 December 2018. Adjusted hazard ratios (AHRs) were calculated for health controls, MCR-non-HGS, and MCR-HGS to predict the onset of dementia and all-cause mortality, initially in an unadjusted model. Adjustments for covariates were made in two stages: Model 1 adjusted for age and gender, while Model 2 further incorporated socioeconomic factors (education level, marital status), lifestyle factors (excessive drinking), and medical conditions (hypertension and diabetes). The predictive accuracy of all models was assessed using discrimination, which is defined by the model’s ability to differentiate between individuals who develop dementia and those who do not, quantified using Harrell’s C-statistic with survival taken into account. To determine the extent of the incremental predictive value added by including HGS (as a continuous variable) to the MCR base model, net reclassification indices (NRI) and integrated discrimination improvement (IDI) were calculated and compared.

Several sensitivity analyses were conducted to verify the stability of our findings. First, to focus on new cases and reduce reverse causation bias, individuals diagnosed with dementia or who died within two years of follow-up were excluded (Sensitivity analysis I). Second, to address missing data, ten imputed data sets were generated using the multiple imputation by chained equations (MICE) method (Wulff and Jeppesen, 2017) for covariates with missing values, and the main analyses were reperformed to check robustness (Sensitivity analysis II). Statistical analyses were conducted using two-tailed tests with a significance level set at *P* < 0.05 and 95% confidence intervals, employing Stata (version 17) for all statistical procedures.

## Results

### Baseline characteristics

The initial characteristics of the study participants are detailed in Table 1. The cohort initially included 5,089 individuals, with prevalence rates of 2.3% for MCR-non-HGS patients and 1.1% for MCR-HGS patients. Among these, MCR-HGS patients were the oldest, with an average age of 77.44 ± 7.37 years and the highest percentage of males at 57.89% in this group. Over the follow-up period, 1,542 patients (30.3%) died. The incidence rates of all-cause dementia were 35.3% for MCR-non-HGS patients and 56.1% for MCR-HGS patients.

**TABLE 1:** Characteristics of included patients at baseline according to MCR status and handgrip strength.

Variable	Non-MCR (*n* = 4913)	MCR patients with normal handgrip strength (*n* = 119)	MCR patients with impaired handgrip strength (*n* = 57)	*p*-value
Age	74.74 ± 6.13	72.62 ± 4.97	77.44 ± 7.37	< 0.001
Male	2, 085 (42.44%)	46 (38.66%)	33 (57.89%)	0.044
**Educational background**				
Illiterate	764 (15.55%)	35 (29.41%)	21 (36.84%)	< 0.001
Primary or above	2, 911 (59.25%)	73 (61.34%)	29 (50.88%)	
Secondary or above	1, 238 (25.20%)	11 (9.24%)	7 (12.28%)	
Married	3, 053 (62.14%)	61 (51.26%)	34 (59.65%)	0.051
**Medical history**				
Hypertension	1, 669 (33.97%)	30 (25.21%)	56 (75.68%)	0.067
Diabetes	1, 081 (22.00%)	38 (31.93%)	11 (19.30%)	0.032
Excessive drink	3, 488 (71.00%)	100 (84. 03%)	43 (75.44%)	0.006
Incident all-cause dementia	814 (16.59%)	28 (23.53%)	20 (35.71%)	< 0.001
Mortality	1, 468 (29.88%)	42 (35.29%)	32 (56.14%)	< 0.001

### Relationships of MCR, MCR-non-HGS patients, and MCR-HGS patients with incident dementia and all-cause mortality

Table 2 shows significant relationships between MCR and MCR-HGS and increased risks of incident dementia, and all models reveal increased risks of all-cause mortality (all *p*-values < 0.001). MCR-HGS had the highest AHRs for both outcomes, with 2.33 (95% CI, 1.49–3.65) for dementia and 1.52 (95% CI, 1.07–2.17) for mortality. MCR-non-HGS patients had a 1.77-fold increased risk of incident dementia (95% CI, 1.21–2.59) when adjusted for age and gender; however, this relationship was not significant when further adjusted for socioeconomic status, lifestyle factors, and medical conditions. Still, a persistent increased risk of all-cause mortality was noted, with a 1.40-fold increase (95% CI, 1.03–1.91) after comprehensive adjustments. For MCR-HGS patients, AHRs for dementia and mortality consistently decreased across models, while for all MCR patients, AHRs initially increased from the unadjusted model to adjusted Model 1, then decreased with further adjustments. Sensitivity analyses I (Supplementary Table 2) and II (Supplementary Table 3) consistently indicated an elevated risk of dementia for MCR and MCR-HGS patients, as well as increased mortality across all groups.

**TABLE 2:** Multivariable analysis for the prediction of dementia and all-cause mortality including baseline characteristics.

	Unadjusted HR^a^ (95% CI)	Model 1^b^: adjusted HR (95% CI)	Model 2^c^: adjusted HR (95% CI)
**All-cause dementia**			
No	Ref.	Ref.	Ref.
MCR patients with normal handgrip strength	1.37 (0.94–2.00)	1.77 (1.21–2.59)	1.33 (0.91–1.95)
MCR patients with impaired handgrip strength	3.21 (2.06–5.01)	2.78 (1.78–4.34)	2.33 (1.49–3.65)
MCR	1.80 (1.34–2.41)	2.09 (1.56–2.80)	1.63 (1.21–2.18)
**All-cause mortality**			
No	Ref.	Ref.	Ref.
MCR patients with normal handgrip strength	1.13 (0.84–1.54)	1.58 (1.16–2.15)	1.40 (1.03–1.91)
MCR patients with impaired handgrip strength	2.32 (1.64–3.30)	1.68 (1.18–2.38)	1.52 (1.07–2.17)
MCR	1.46 (1.15–1.84)	1.62 (1.28–2.05)	1.33 (1.05–1.68)

^a^HR, hazard ratio.

^b^Model 1 adjusted for age and gender.

^c^Model 2 further adjusted for educational background, marital status, excessive drinking, hypertension and diabetes.

### Added value of HGS to MCR syndrome in predicting incident dementia and all-cause mortality

Compared with MCR alone, the discrimination (C-statistic) for predicting incident dementia and all-cause mortality is higher when HGS is included (MCR+HGS), as shown in Table 3. The C-statistic significantly increased from 0.7142 for MCR alone to 0.7194 for MCR+HGS in predicting incident dementia (*p*-value = 0.023), and from 0.7114 for MCR alone to 0.718 for MCR+HGS in predicting all-cause mortality (*p*-value < 0.001). Additionally, incorporating HGS into the multivariate model improves discrimination (IDI = 0.0022 for incident dementia and 0.01 for all-cause mortality, *p*-value < 0.01). The NRI also underscores this improved discrimination when continuous HGS is added to MCR prediction models (NRI = 0.0751 for incident dementia, *p*-value = 0.0445; NRI = 0.1735 for all-cause mortality, *p*-value < 0.0001).

**TABLE 3:** Modification of the predictive value after adding the handgrip strength to the MCR syndrome.

	Adjusted HR^a^ (95% CI)^b^	C-statistic	Δ C-statistic	*p*-value	IDI^c^	*p*-value	NRI^d^	*p*-value
**All-cause dementia**								
MCR	1.63 (1.21–2.19)	0.7142						
MCR+handgrip strength	1.53 (1.14–2.06)	0.7194	0.0052	0.023	0.0022	0.0036	0.0751	0.0445
**All-cause mortality**								
MCR	1.45 (1.15–1.84)	0.7114						
MCR+handgrip strength	1.33 (1.05–1.68)	0.718	0.0066	< 0.001	0.01	< 0.0001	0.1735	< 0.0001

^a^HR, hazard ratio.

^b^95% CI, 95% confidence interval.

^c^IDI, integrated discrimination improvement.

^d^NRI, net reclassification indices.

**FIGURE 2:**
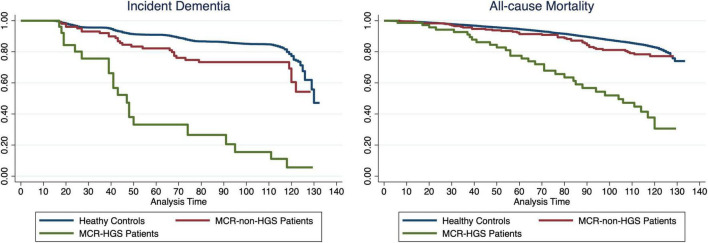
Kaplan-Meier Survival Curve showing the proportion of dementia-free or survival participants during follow-up between MCR-non-HGS, MCR-HGS patients and healthy controls.

The Kaplan–Meier curve, depicted in Figure 2, illustrates the duration to incident dementia or all-cause mortality, stratified by MCR-non-HGS, MCR-HGS, and healthy controls, with adjustments made for all covariates. Both curves show a decline over the follow-up period, with a notably pronounced decrease observed among patients (all *p*-values for log-rank tests < 0.0001).

## Discussion

To our knowledge, this is the first study to assess the benefit of integrating HGS with MCR in predicting incident dementia and all-cause mortality among a large, representative cohort of older adults across a follow-up period exceeding 10 years. Our results corroborate previous studies on the accuracy of MCR in estimating dementia risk, despite variations in study populations, follow-up lengths, and definitions of MCR and dementia diagnosis. We demonstrate the discriminative and predictive power of the MCR-HGS combination in forecasting future adverse health outcomes, thereby enabling the early identification of older adults in need of further clinical evaluation and those at increased risk of developing dementia. Additionally, we found that MCR patients with normal handgrip strength do not show a heightened risk of incident dementia following comprehensive adjustment, highlighting the improved clinical utility and applicability of the MCR-HGS approach, especially in environments where handgrip strength assessment is practical.

The AHR for all-cause dementia among MCR-HGS patients (2.33; 1.49–3.65) was comparable to that reported for MCR in a multicohort study (AHR = 1.93) (Verghese et al., 2014), and exceeded the rates found for other modified MCR concepts such as MCR-TUG (timed-up-and-go test, AHR = 2.03) and MCR-OLS (one-leg-standing test, AHR = 2.05) from previous studies (Chung and Byun, 2023). However, we found no significant associations between MCR-non-HGS and incident dementia after adjusting for all confounders, which suggests that diminished muscle strength might act as an early marker of impaired neural processing (Alfaro-Acha et al., 2006), offering greater sensitivity and precision in measuring cognitive function than subjective cognitive decline. In contrast, an MCR subtype characterized using the 5-times-sit-to-stand, which includes a balance component, was less predictive of cognitive decline than MCR defined by slow gait in earlier studies (Sekhon et al., 2019b). The complex nature of maximum grip strength, which requires intricate coordination of numerous motor units and brain networks, was highlighted (Jia et al., 2023). Previous research has also connected MCR with reduced gray matter volume, particularly in regions such as the premotor cortex and prefrontal areas, alongside lacunar lesions in the frontal lobe, indicating a better capability to predict neurodegenerative cortical dementia over subcortical types (Beauchet et al., 2016; Sekhon et al., 2019a). Moreover, neural networks associated with MCR displayed atrophy in gray matter areas involved in gait control, particularly in planning and modulation, rather than in motor execution. Yet, detailed studies on the structural relationships between HGS and MCR within the central nervous system are still scarce. Considering the ease of measuring HGS, MCR-HGS could be advocated as a valuable diagnostic tool for predicting adverse outcomes in older adults. More research is necessary to determine the pathological importance of HGS and to clarify the prognostic significance of MCR-HGS in forecasting future all-cause dementia and mortality (Blumen et al., 2019).

In our study, the AHR for all-cause mortality among MCR-HGS patients was 1.52 (95% CI 1.07–2.17), a magnitude similar to those reported for MCR syndrome (Ayers and Verghese, 2016), moderate-to-severe cognitive impairment (Kelman et al., 1994; Sachs et al., 2011; Perna et al., 2014), and other predementia syndromes (Gussekloo et al., 1997; Park et al., 2014). In contrast to incident dementia, a consistent and significant association was observed between MCR-non-HGS and all-cause mortality across all models. Specifically, the mortality risks were 1.58 (95% CI 1.16–2.15) and 1.40 (95% CI 1.03–1.91) in adjusted Models 1 and 2, respectively. Predementia syndromes may elevate mortality risk by exacerbating geriatric syndromes that are associated with higher mortality in the aging population. For instance, cognitive impairment can increase the risk of life-threatening events such as delirium (Inouye, 2006), depression (Pellegrino et al., 2013), medication mismanagement (Hayes et al., 2009), and falls (Doi et al., 2015). Previous studies have shown that MCR is associated with a higher risk of developing Alzheimer’s disease dementia and vascular dementia subtypes (Verghese et al., 2013, 2014). Moreover, pathologies related to dementia, including cerebrovascular disease and regional brain atrophy particularly in the frontal lobes, have been linked to increased mortality risks (Nägga et al., 2014).

MCR-HGS shows incremental predictive validity for all-cause mortality beyond that of MCR alone in our study. While both low HGS and slow gait speed are associated with a higher risk of mortality (Cooper et al., 2010), previous studies have not investigated the combined effects of HGS, gait speed, and subjective cognitive complaints on mortality. Several possible reasons can explain this observation. First, muscle weakness associated with aging may indicate chronic disease (Guadalupe-Grau et al., 2015) and a decline in physical function (Bohannon, 2008; Bouchard et al., 2009), both of which are connected to a higher risk of mortality (Yerrakalva et al., 2015; Pavasini et al., 2016). Second, changes in HGS can more quickly reflect nutritional deficiencies or recovery compared to alterations in muscle mass (Norman et al., 2011). Malnutrition can heighten mortality risk, with changes in related biomarkers potentially making elderly patients more susceptible to infections and associated mortality (Yoshikawa and High, 2001; Norman et al., 2011). Third, simultaneous declines in cognitive and physical capabilities correlate with reduced hemoglobin levels (Atkinson et al., 2005), which may directly reduce oxygen delivery to the brain, peripheral nerves, and muscles. Moreover, poor physical performance is linked to significant endocrine dysfunction, inflammation, and oxidative stress, all factors that increase mortality risk (Cooper et al., 2010).

This study supports the validity of modified MCR concepts (MCR-HGS) as estimators of dementia and mortality in a nationally representative, homogeneous population, following adjustments for age-related confounding factors. Although neurophysiologic tests are generally expensive and require specialized professionals for administration, MCR-HGS provides a simple and efficient alternative for identifying high-risk individuals. Using continuous measurements minimizes information loss, and dichotomized variables for HGS can be derived from population-based cut-points (Bahat et al., 2020) or previous guidelines (Blanquet et al., 2022). Furthermore, the MCR-HGS assessment is not influenced by the participant’s educational level or by learning effects from repeated testing, which enhances its credibility and validity. Nevertheless, the approach has several limitations. Additional HGS testing, while potentially increasing the ability to discriminate negative health outcomes, also adds to the physicians’ workload and limits the feasibility of remote assessments due to the need for dynamometers. The lack of objective neuropsychological testing may lead to the oversight or misdiagnosis of some conditions. However, a sensitivity analysis that excluded participants diagnosed with dementia within two years of the index date strengthened the robustness of our findings. Moreover, due to differences in individual HGS profiles across various countries, our conclusions may not be generalizable to other populations. Future research involving multi-country cohorts is warranted to validate our findings across diverse populations. Finally, it was not possible to control for other confounding variables that may influence dementia development, such as APOE genotype or imaging biomarkers (Baumgart et al., 2015), in this study.

## Conclusion

Our extensive nationwide cohort study demonstrates the added value of handgrip strength in the modified MCR (MCR-HGS) for predicting incident dementia and all-cause mortality, beyond the original MCR concepts. These results indicate that modified MCR can act as an effective and practical screening method to estimate the risks of dementia and mortality during national health assessments in older populations. Future research should explore the cost-effectiveness of incorporating HGS measurements in clinical or community settings versus reliance on self-reported questionnaires to identify potential patients. Additionally, further studies on the physical and neurobiological characteristics of MCR-HGS as a risk factor for dementia and mortality are needed, along with analyses of how MCR combined with other physical capabilities might better identify at-risk individuals.

## Data availability statement

The datasets presented in this study can be found in online repositories. The names of the repository/repositories and accession number(s) can be found below: https://hrsdata.isr.umich.edu/data-products/public-survey-data.

## Ethics statement

The studies involving humans were approved by the University of Michigan Institutional Review Board approved the HRS study. The studies were conducted in accordance with the local legislation and institutional requirements. The participants provided their written informed consent to participate in this study.

## Author contributions

WB: Conceptualization, Data curation, Methodology, Supervision, Validation, Visualization, Writing – original draft. RM: Formal analysis, Methodology, Software, Validation, Visualization, Writing – review & editing. YY: Formal analysis, Methodology, Supervision, Validation, Visualization, Writing – review & editing. JX: Data curation, Funding acquisition, Project administration, Supervision, Visualization, Writing – review & editing. LQ: Conceptualization, Data curation, Methodology, Supervision, Validation, Visualization, Writing – review & editing.
