# Supplementary material for: Enhancing predictive validity of motoric cognitive risk syndrome for incident dementia and all-cause mortality with handgrip strength: insights from a prospective cohort study
Source: Front Aging Neurosci. 2024 Jun 21;16:1421656. doi: 10.3389/fnagi.2024.1421656 (PMC11224449; doi:10.3389/fnagi.2024.1421656)
Supplement: Supplementary file 1 [file Table_1.docx]

**Supplement Materials**

**Table S1. Operational definition of slow gait and weakness among studied population.**

**Table S2. Associations between MCR subtype, incident dementia and all-cause mortality after excluding the participants developing dementia or deceased in the first two year.**

**Table S3. Associations between MCR, handgrip strength, incident dementia and all-cause mortality after imputation.**

**Table S1.** Operational definition of slow gait and weakness among studied population.

| **Criteria** | **Definition** |  |  |  |  |
| --- | --- | --- | --- | --- | --- |
| **Slowness** | **Gait Speed (Men)** |  | **Gait Speed (Women)** | | |
|  | Age < 75 years | 0.61 m/s | Age < 75 years | 0.54 m/s |  |
|  | Age ≥75 years | 0.48 m/s | Age ≥75 years | 0.42 m/s |  |
| **Weakness** | **Grip Strength (Men)** | | **Grip Strength (Women)** | | |
|  | BMI < 18.5 kg/m^2^ | 19.5 kg | BMI < 18.5 kg/m^2^ | 14.25 kg |  |
|  | BMI 18.5 - 24.9 kg/m^2^ | 27.38 kg | BMI 18.5 - 24.9 kg/m^2^ | 16.25 kg |  |
|  | BMI 24.9 - 29.9 kg/m^2^ | 29.4 kg | BMI 24.9 - 29.9 kg/m^2^ | 17 kg |  |
|  | BMI > 29.9 kg/m^2^ | 30.28 kg | BMI > 29.9 kg/m^2^ | 18.5 kg |  |

**Table S2.** Associations between MCR subtype, incident dementia and all-cause mortality after excluding the participants developing dementia or deceased in the first two year.

|  | Unadjusted HR  (95%CI) | Model 1: adjusted HR (95%CI) | Model 2:  adjusted HR  (95%CI) | |
| --- | --- | --- | --- | --- |
| Incident All-cause Dementia | | |  |  |
| No | Ref. | Ref. | Ref. |  |
| MCR patients with normal handgrip strength | 1.38  (0.88 - 2.15) | 1.81  (1.16 - 2.84) | 1.40  (0.89 - 2.20) | |
| MCR patients with impaired handgrip strength | 3.75  (2.20 - 6.39) | 3.39  (1.99 - 5.77) | 2.94  (1.73 - 5.01) | |
| MCR | 1.86  (1.32 - 2.63) | 1.86  (1.32 - 2.63) | 2.25  (1.59 - 3.18) | |
| All-cause Mortality | |  |  |  |
| No | Ref. | Ref. | Ref. |  |
| MCR patients with normal handgrip strength | 1.15  (0.84 - 1.58) | 1.65  (1.29 - 2.10) | 1.41  (1.02 - 1.95) | |
| MCR patients with impaired handgrip strength | 2.36  (1.63 - 3.40) | 1.60  (1.16 - 2.20) | 1.55  (1.07 - 2.25) | |
| MCR | 1.47  (1.15 - 1.88) | 1.72  (1.19 - 2.48) | 1.47  (1.15 -1.88) | |

**Table S3.** Associations between MCR, handgrip strength, incident dementia and all-cause mortality after imputation.

|  | Unadjusted HR  (95%CI) | Model 1: adjusted HR (95%CI) | Model 2:  adjusted HR  (95%CI) | |
| --- | --- | --- | --- | --- |
| Incident All-cause Dementia | | |  |  |
| No | Ref. | Ref. | Ref. |  |
| MCR patients with normal handgrip strength | 1.37  (0.94 - 1.99) | 1.77  (1.21 - 2.58) | 1.33  (0.91 - 1.94) | |
| MCR patients with impaired handgrip strength | 3.20  (2.05 - 5.00) | 2.78  (1.78 - 4.33) | 2.33  (1.49 - 3.64) | |
| MCR | 1.79  (1.34 - 2.40) | 2.08  (1.56 - 2.79) | 1.62  (1.21 - 2.18) | |
| All-cause Mortality | |  |  |  |
| No | Ref. | Ref. | Ref. |  |
| MCR patients with normal handgrip strength | 1.14  (0.84 - 1.54) | 1.58  (1.16 - 2.15) | 1.40  (1.03 - 1.91) | |
| MCR patients with impaired handgrip strength | 2.33  (1.64 - 3.30) | 1.68  (1.18 - 2.38) | 1.53  (1.07 - 2.18) | |
| MCR | 1.46  (1.16-1.84) | 1.62  (1.28 - 2.05) | 1.46  (1.15 - 1.84) | |
